# Prediction of 2 years-survival in patients with stage I and II non-small cell lung cancer utilizing ^18^F-FDG PET/CT SUV quantification

**DOI:** 10.2478/raon-2013-0023

**Published:** 2013-07-30

**Authors:** Angelina Cistaro, Natale Quartuccio, Alireza Mojtahedi, Piercarlo Fania, Pier Luigi Filosso, Alfredo Campenni, Umberto Ficola, Sergio Baldari

**Affiliations:** 1 Positron Emission Tomography Centre IRMET S.p.A., Euromedic inc., Turin, Italy; 2 Nuclear Medicine Unit, Department of Biomedical Sciences and of the Morphological and Functional Images, University of Messina, Messina, Italy; 3 Nuclear Medicine Service, Memorial Sloan-Kettering Cancer Center, New York, USA; 4 Department of Thoracic Surgery, S. Giovanni Battista Hospital, Turin, Italy; 5 Department of Nuclear Medicine, La Maddalena Hospital, Palermo, Italy

**Keywords:** 2-deoxy-2-[18F]fluoro-D-glucose positron emission tomography, non-small cell lung cancer, maximum standardized uptake value, disease-free survival, overall survival

## Abstract

**Background:**

The purpose of the study was to evaluate the correlation between the maximum standardized uptake value (SUVmax), size of primary lung lesion, disease-free survival (DFS) and overall survival (OS) in patients with stage I and II non-small cell lung cancer (NSCLC) in 2 years follow-up.

**Patients and methods.:**

Forty-nine patients with stage I–II NSCLC were included in this study. Pre-surgical 2-deoxy-2-[18F]fluoro-D-glucose positron-emission tomography (^18^F-FDG PET/CT) study was performed for all patients. The relationship between SUVmax, tumour size and clinical outcome was measured. The cut-off value for SUVmax and tumour size with the best prognostic significance, probability of DFS and the correlation between SUVmax and the response to therapy were calculated.

**Results:**

There was a statistically significant correlation between SUVmax and DFS (p = 0.029). The optimal cut-offs were 9.00 for SUVmax (p = 0.0013) and 30mm for tumour size (p = 0.0028). Patients with SUVmax > 9 and primary lesion size > 30 mm had an expected 2years-DFS of 37.5%, while this rose to 90% if the tumour was <30 mm and/or SUVmax was <9.

**Conclusions:**

In stage I-II, SUVmax and tumour size might be helpful to identify the subgroup of patients with high chance for recurrence.

## Introduction

Lung cancer (LC) is the major cause of death in the developing countries, with an incidence of about 65–70 new cases per 100.000.^1^ 2-deoxy-2-[18F] fluoro-D-glucose positron-emission tomography (^18^F-FDG PET/CT) is widely used in lung cancer for staging, restaging and evaluation of the treatment response.^2^,^3^ Multiple studies demonstrate that PET/CT is more sensitive and specific than PET alone in evaluating the lung cancer since it provides combined morphological and functional information of the tumour.^4^–^7^ High accuracy of PET/CT has been observed in the early assessment of response to therapy, showing a close correlation between the reduction of tumour metabolic activity measured after a course of therapy and the clinical outcome of patients after the previewed cycles of therapy in patients in advanced stage.^8^–^9^ In early stage, Tumour Node Metastasis (TNM) staging system, it is still the most reliable prognostic factor to predict the outcome after surgery. However, many patients in stage I or II may experience a worse outcome than expected and an early relapse even after a successful tumour resection.^10^ Also magnetic resonance imaging (MRI) 11, computed tomography (CT) 12 and endobronchial ultrasonography (EBUS)^13^ have a role in the staging of lung cancer but, unlike PET/CT, lack in providing functional information, which may provide clinicians additional prognostic indications regarding the survival and the estimate risk of relapse by means of the assessment of the maximum standardized uptake value (SUVmax).

The aim of this study was to estimate the cut-off value for combined SUVmax and size of primary lung lesion that correspond with poor disease-free survival (DFS) in patients with stage I and II non-small cell lung cancer (NSCLC) in 2 years follow-up.

## Patients and methods

Between 2004 and 2009, forty-nine patients (36 males = 73%; 13 females = 27%; M/F Ratio = 2.77:1; mean age = 67.2 years; range = 44–79 years) were referred to Positron Emission Tomography Centre IRMET S.p.A., Euromedic inc. in Turin, Italy and Unit of Nuclear Medicine of La Maddalena Hospital in Palermo, Italy for the evaluation of their non-small cell lung cancer. According to the inclusion criteria 49 patients included in this study received a complete tumour resection and systematic lymph node dissection.

All patients have been staged according to TNM 7^th^ edition.^14^ Survival and death information were obtained from the hospitals databases and through phone calls to the patient families. The research proposal was approved by Institutional Review Board and Ethics Committee.

The inclusion criteria were histologically proven NSCLC, glycaemia lower than 140 mg/dl at the time of the exam, availability of pre-surgical FDG-PET/CT and tumour size > 20 mm to minimize the underestimation of SUV. Exclusion criteria were as follows: (a) poor performance status; (b) diabetes (due to poor uptake of FDG); (c) pregnancy; (d) Charlson Combined Age-Comorbidity Index ≥ 6; (e) histological diagnosis of “bronchioloalveolar cell carcinoma” (BAC) subtype.

Preoperative TNM staging was obtained via information gathered through patient’s chart including physical examination, routine blood test, bronchoscopy, contrast-enhanced CT of the chest and upper abdomen, brain CT and total-body ^18^F-FDG PET/CT scan.

The PET/CT images were acquired using integrated PET/CT scanner (Discovery ST, General Electric Medical System, Milwaukee, WI, USA), 60 minutes after the injection of the 260–420 MBq of ^18^F-FDG, following 6 hours of fasting from the cranial base to the pelvis (total body) in 3D modality. Blood glucose was measured before the injection of the tracer to ensure glucose blood levels < 140 mg/dL. The PET images were reconstructed iteratively on a 128 × 128 matrix. The SUVmax, within a spherical region of interest encompassing the entire volume, was calculated using a dedicated workstation (Xeleris, General Electric medical Systems, Milwaukee, WI, USA). The formula used for SUV calculation was: activity (MBq/ml) x body weight / injected dose (MBq/ml). The anatomical size of lung lesion was measured considering the maximum diameter of the lesion in the three planes on CT.^15^

PET, CT, and fused PET/CT images were reviewed, by two experienced nuclear medicine physicians. Images were assessed visually and semi-quantitatively. Patients were followed up for 24 months with a frequency of clinical examination of every 3 months during the first year and every 6 months in the second year. A contrast-enhanced CT scan of the thorax was performed in all cases at 6 and 12 months, while the additional PET/CT scans were used in case of suspected morphological findings.

The overall survival (OS) was calculated. The disease-free survival (DFS) was considered as the time between surgery and recurrence of disease. The correlation between SUVmax and clinical outcome (2 years-DFS) was assessed by the Student-t test (the hypothesis was considered significant if p </= 0.05). The chi-square test was used to identify the SUVmax cut-off value at the time of the initial staging with the best correlation to prognosis.

Finally, the estimated probability of disease-free time according to the SUVmax cut-off and the dimensional threshold was calculated using the Kaplan-Meier method.

## Results

Twenty-eight out of 49 patients (M = 36, F = 13, M/F Ratio = 2.77: 1; mean age = 67.2 years, range: 44–79) diagnosed with adenocarcinoma (BAC excluded according to exclusion criteria) which was the most frequent tumour histologic subtype (57%). Squamous cell carcinoma was observed in 15 patients (31%), and large cell carcinoma in 2 cases (4%). In 4 patients (8%) no sufficient data about pathological examination were available and they were classified as undetermined (Table 1).

At the time of the pre-surgical PET/CT scan, the patients in stage I–II showed an average SUVmax value of 10 ± 5.5 and an average size of primary lung lesion of 33 mm ± 17.00; at the follow-up period of 24 months, DFS and OS were respectively 73.4% and 83,6 %. 36 patients (average SUVmax value of 8.96 ± 5.26; mean size of primary lung lesions of 31.27 mm ± 18.65) were 2 years disease-free and presented an OS of 100%. 13 patients (average SUVmax = 12.8 ± 5.3; mean size of primary lung lesion = 40.5 ± 9.4 mm) had recurrence of disease (median DFS = 10 months, OS = 39.5%). There was a statistically significant correlation between SUVmax and DFS (p = 0.029, Student-t test). Using the chi-square test, the SUVmax cut-off and the dimensional cut-off with the best prognostic significance were 9.00 (p = 0.0013) and 30 mm (p = 0.0028) respectively. In the subgroup of 26 patients presenting with a primitive lung lesion larger than 30 mm, there were 12 recurrence of disease events (Table 2A–D), 2 of them with a SUV max <9 and 10 of them with a SUV max > 9.

Combining metabolic and dimensional parameters, patients with SUV > 9 and primary lesion size >30 mm had an expected 2 years-DFS of 37.5%, while the expected 2 years-DFS raised to 90% if the tumour was smaller than 30 mm and/or the SUVmax was below 9.00 (one or both cut-offs) (Table 2A–D).

**FIGURE 1.**
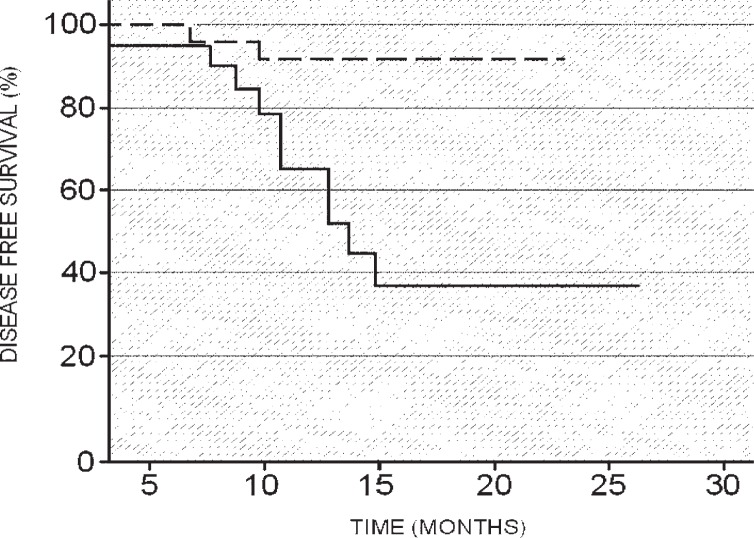
Disease-free survival (DFS) curves (Kaplan-Meier) based on a dimensional cut-off of 30 mm. The disease-free survival curves illustrate that patients with a lung cancer with dimension < 30 mm present a better prognosis (significant longer DFS) than patients with lung cancer size > 30 mm. Dashed line = DFS for patients in stage I–II with lesion diameter < 30 mm. Continuous line = DFS for patients in stage I–II with lesion diameter > 30 mm.

**FIGURE 2.**
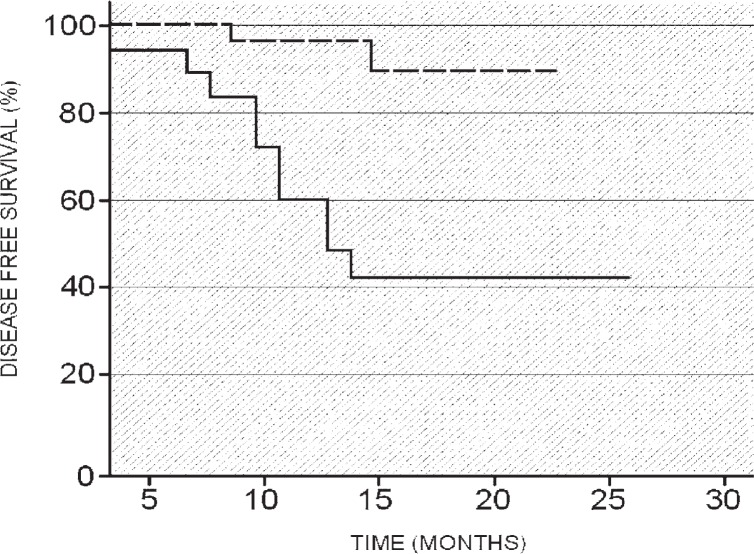
Disease-free survival (DFS) curves (Kaplan-Meier) based on a cut-off maximum standardized uptake value (SUVmax) of nine. The disease-free survival curves illustrate that patients with lung cancer SUVmax < 9 present a significant longer DFS than patients with lung cancer SUVmax > 9. Dashed line = DFS for patients in stage I–II with SUVmax < 9; Continuous line = DFS for patients in stage I–II with SUVmax > 9.

With cut-off values (SUVmax = 9, size = 30 mm) and using the univariate statistical analysis according to the Kaplan-Meier method, two different disease-free survival curves were generated (Figures 1, 2). The analysis of the disease-free survival curves illustrated that patients with SUVmax < 9 and patients with a primary lung cancer size below 30 mm demonstrated a significant longer DFS (Figure 3A,B) in comparison with patients with SUVmax > 9 or patients with a primitive lesion > 30 mm.

## Discussion

Among several prognostic staging systems of NSCLC, the TNM system still remains the most widely used. Despite this, the clinical outcome doesn’t always follow the clinical prognostic assessment, so the disease can sometimes result in more unfavorable outcome than expected.^2^,^16^ Even if a complete surgical resection is accomplished, NSCLCs can relapse. These data are extremely dramatic in patients in stage I and II, in whom recurrences of disease will happen and a more aggressive treatment would have been beneficial.^2^ In NSCLC, the use of SUV measurements previously reported to be accurate and reproducible in the assessment of the outcome.^17^–^22^

In our study with patients in stage I–II it has been attempted to focus on the prognostic significance of SUVmax and dimensional parameter of the primitive neoplastic lung lesion in reference with the 2 years disease-free survival. In the present study we accurately selected patients undergoing only the surgical procedure. The cut-off value of a SUVmax in a 2 years follow-up period has not been clearly defined in literature.^23^–^28^ Jeong *et al.* determined a cut-off value of 7 to be indicator of poor disease-free survival (DFS) 24, while Goodgame and colleagues found the threshold value of SUV to be 5.5.^12^ Our data showed that in stages I and II, the increased size more than threshold (30 mm) and SUVmax cut-off (9.00) could help to identify a subgroup of patients with a high risk of recurrence within 2 years after curative surgery. The relative estimated risk for patients with SUVmax < 9 and/or tumour size < 30 mm is lower (10%) (p = 0.004) compared to the group above these cut-offs. Similar results had been documented by the Leuven Lung Cancer Group which reported a 2-year survival rate of 86% if the resected tumour was smaller than 3 cm and the SUV was below 7.^27^

Our data showed that, in the early stages of disease, SUVmax and dimension of the primitive lesion were independent prognostic factors and this correlated with 3 previously published papers.^11^,^25^,^28^ In our series, the additional prognostic information provided by the SUVmax cut-off showed the most powerful impact in case of tumour > 30 mm. In fact the highest rate of recurrence (62.5%) was recorded in case of tumour SUVmax >9 and size >30 mm (10 recurrence of disease and 6 disease-free patients), while patients with SUVmax <9 and tumour dimension >30 mm, presented a lower rate of recurrence (20%; 8 disease –free and 2 recurrence of disease events). However, due to the small number of patients it was not possible to attempt to demonstrate any statistical correlation.

Our study, however, had several limitations including a limited number of patient, lack of SUV quantification in each single histotype, and different cut off value for each subtype of NSCLC. Additional studies are required to further accurately assess the correlation between SUVmax and size of primary lung lesion with DFS and OS in different histotypes in 2 years period.

## Conclusions

Our data suggested that in patients with NSCLC stage I–II the combination of a dimensional cut-off (30 mm) and of a SUVmax cut-off (9) could identify a subgroup of patients that would have a high risk of recurrence of disease within 2 years from surgery.

## Figures and Tables

**FIGURE 3A.**
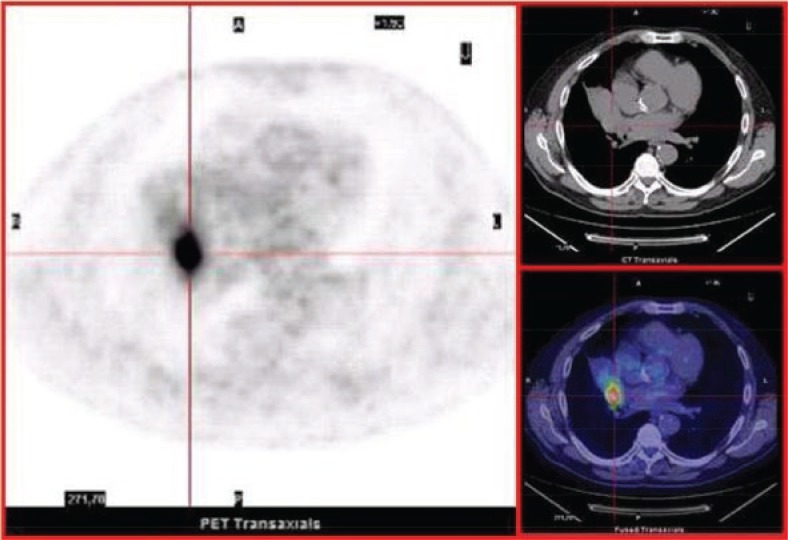
Pre-surgical ^18^F-FDG PET/CT axial images. Abnormal 18F-FDG uptake (SUVmax < 9) in a cancer lesion located in the perihilar region of the right lung (size < 30 mm).

**FIGURE 3B.**
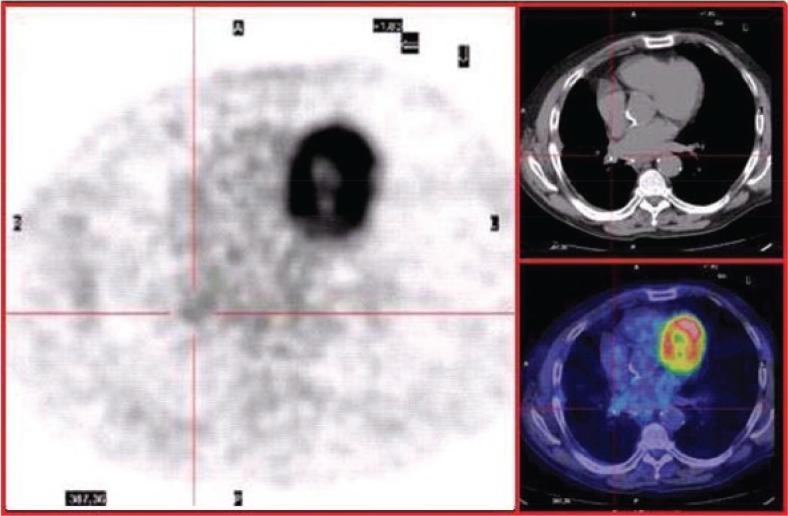
Post-surgical ^18^F-FDG PET/CT axial images. No pathological ^18^F-FDG uptake can be detected in the perihilar region of the right lung.

**TABLE 1.** Characteristics of population

**Number of Patients**	**49**	
Sex	36 M	13 F
Mean Age	67.2 y (range: 44–79)	
Histotypes:		
Adenocarcinoma	28	
Squamous	15	
Large cell	2	
Undetermined	4	

**TABLE 2A.** Summary of (stage I–II) patients’ outcome at a follow-up period of 24 months

**DFS**	36 disease free	13 recurrence of disease
**OS**	41 alive	8 dead

**TABLE 2B.** Stratification of disease-free survival for the maximum standardized uptake value (SUVmax) cut-off at a follow-up period of 24 months. (Chi-square test) p = 0.0013

**DFS**	**SUVmax < 9**	**SUVmax > 9**
Disease-free	26	10
Recurrence of disease	2	11

**TABLE 2C.** Stratification of disease-free survival for a size threshold of 30 mm at a follow-up period of 24 months. (Chi-square test) p = 0.0028

**DFS**	**Size < 30 mm**	**Size > 30 mm**
Disease-free	22	14
Recurrence of disease	1	12

**TABLE 2D.** Stratification of disease-free survival for the combined maximum standardized uptake value (SUVmax) and size cut-offs at a follow-up period of 24 months. (Chi-square test) p = 0.0003

**DFS**	**SUV max < 9 and/or Size < 30 mm**	**SUV max > 9 and Size > 30 mm**
Disease-free	30	6
Recurrence of disease	3	10
